# Computational Drug Repositioning and Experimental Validation of Ivermectin in Treatment of Gastric Cancer

**DOI:** 10.3389/fphar.2021.625991

**Published:** 2021-03-31

**Authors:** Hanne-Line Rabben, Gøran Troseth Andersen, Aleksandr Ianevski, Magnus Kringstad Olsen, Denis Kainov, Jon Erik Grønbech, Timothy Cragin Wang, Duan Chen, Chun-Mei Zhao

**Affiliations:** ^1^ Department of Clinical and Molecular Medicine, Norwegian University of Science and Technology (NTNU), Trondheim, Norway; ^2^ The Central Norway Regional Health Authority (RHA), Stjørdal, Norway; ^3^ Surgical Clinic, St. Olavs Hospital, Trondheim University Hospital, Trondheim, Norway; ^4^ Division of Digestive and Liver Diseases, Columbia University College of Physicians and Surgeons, New York, NY, United States

**Keywords:** gastric cancer, ivermectin, drug repositioning, ingenuity pathway analysis, *in silico*

## Abstract

**Objective:** The aim of the present study was repositioning of ivermectin in treatment of gastric cancer (GC) by computational prediction based on gene expression profiles of human and mouse model of GC and validations with *in silico*, *in vitro* and *in vivo* approaches.

**Methods:** Computational drug repositioning was performed using connectivity map (cMap) and data/pathway mining with the Ingenuity Knowledge Base. Tissue samples of GC were collected from 16 patients and 57 mice for gene expression profiling. Additional seven independent datasets of gene expression of human GC from the TCGA database were used for validation. *In silico* testing was performed by constructing interaction networks of ivermectin and the downstream effects in targeted signaling pathways. *In vitro* testing was carried out in human GC cell lines (MKN74 and KATO-III). *In vivo* testing was performed in a transgenic mouse model of GC (INS-GAS mice).

**Results:** GC gene expression “signature” and data/pathway mining but not cMAP revealed nine molecular targets of ivermectin in both human and mouse GC associated with WNT/β-catenin signaling as well as cell proliferation pathways. *In silico* inhibition of the targets of ivermectin and concomitant activation of ivermectin led to the inhibition of WNT/β-catenin signaling pathway in “dose-depended” manner. *In vitro*, ivermectin inhibited cell proliferation in time- and concentration-depended manners, and cells were arrested in the G_1_ phase at IC_50_ and shifted to S phase arrest at >IC_50_. *In vivo*, ivermectin reduced the tumor size which was associated with inactivation of WNT/β-catenin signaling and cell proliferation pathways and activation of cell death signaling pathways.

**Conclusion:** Ivermectin could be recognized as a repositioning candidate in treatment of gastric cancer.

## Introduction

Drug repositioning (also called drug repurposing) is a strategy for identifying new uses for approved or investigational drugs that are outside the scope of the original medical indications. Repositioned drugs may reveal new targets and pathways that can be further exploited (Ashburn and Thor, 2004; Pushpakom et al., 2019). Advantages of drug repositioning are related to the drugs that have known mechanisms of action, pharmacological properties, such as pharmacokinetics, pharmacodynamics, posology (the appropriate doses of drugs) and toxicity (Verbaanderd et al., 2017). Of note is that both the pre-clinical and clinical safety data are available (Verbaanderd et al., 2017; Antoszczak et al., 2020). Thus, compared to traditional methods of drug development, drug repositioning requires drastically shortened development time and reduced costs while providing similar therapeutic benefits. Approaches of drug repositioning include computational methods, such as connectivity map (cMap), data mining, pathway mining, ontology modeling, *in silico* and biological experimental validations (e.g., *in vitro* and *in vivo*). The computational drug repositioning can be conducted as repurposing with a defined purpose, repurposing with a strategy, and repurposing with confidence by utilizing reference datasets which are disease-based, drug-based, or knowledge-based (Liu et al., 2013; Subramanian et al., 2017; Xue et al., 2018).

Ivermectin was identified in late 1960s, first approved as veterinary medicine and then human medicine in 1980s for the control of parasitic infection. The discovery and development of ivermectin by William C. Campbell and Satoshi Ōmura were recognized by Nobel Prize in Physiology or Medicine in 2015 (Callaway and Cyranoski, 2015). Ivermectin acts on γ-aminobutyric acid (GABA)-gated chloride channels in the interneuronic synapses of a parasite, whereas in humans, the nerves that are sensitive to GABA, are protected by the blood/brain barrier (Sutherland and Campbell, 1990; Davis et al., 1999; Ikeda, 2003; Chen and Kubo, 2018). However, ivermectin has been repositioned as a broad-spectrum antiviral and antimicrobial agent (Andersen et al., 2020). Interestingly, it is also known that ivermectin can be widely distributed in humans because of its high lipophilicity and thus might exhibit anti-tumor activity in colorectal cancer, breast cancer, glioma, head and neck cancer, leukemia, melanoma, pancreatic cancer, and prostate cancer (Chiou et al., 1987; Juarez et al., 2018).

Gastric cancer (GC) is the fifth most common malignant disease worldwide with the third highest incidence and mortality rate among all cancers (Rawla and Barsouk, 2019). The 5 years overall survival rate is 10–30% except for Japan (50–70%) (Parkin et al., 2002; Matsuda and Saika, 2013). Gastrectomy combined with platinum-based chemotherapy is the most beneficial approach in patient care, and novel targeted therapy, including PD-1 inhibitor in first and second-line setting for advanced GC, are under development (Sitarz et al., 2018; Selim et al., 2019; GBD 2017 Stomach Cancer Collaborators, 2020). However, new drugs and drug repositioning are needed particularly in consideration of the global burden of this deadly disease.

**FIGURE 1:**
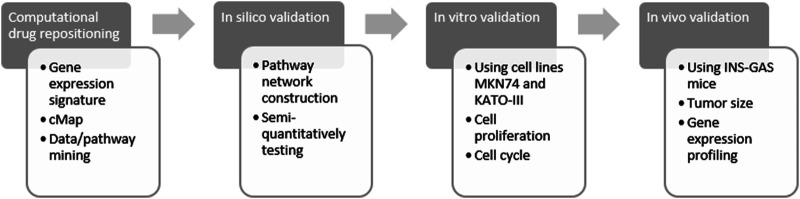
Flow chart of study design. Computational drug repositioning was carried out by using gene expression signatures representing gastric cancer of both patients and mouse model and connectivity map (cMap) and data/pathway mining with the Ingenuity Knowledge Base. Validation included *in silico*, *in vitro* and *in vivo* methods of ivermectin treatment. The rationale of using human samples of GC (without ivermectin treatment) was *i*) to perform computational prediction and data mining and *ii*) to make a comparison with the animal model in order to demonstrate that the animal study could be relevant in the design of clinical trial of ivermectin in the future.

Previously, we have showed repositioning of botulinum toxin type A (also known as botox), everolimus (RAD001) and devimistat (CPI-613) in treatment of GC (Zhao et al., 2014; Rabben et al., 2016; Rabben et al., 2021). The aim of the present study was to reposition ivermectin in treatment of GC. To this end, we have developed and/or utilized the approaches from computational drug repositioning to *in silico*, *in vitro* and *in vivo* validations (Figure 1).

## Materials and Methods

### Patients and Animals

Surgical biopsies were collected from 16 patients who underwent total/subtotal or distal gastrectomy because of GC since 2012 at St. Olav’s Hospital, Trondheim, Norway (Table 1). Four biopsies per patients were taken from tumor and normal tissue and used for clinical pathological evolution and gene expression profiling. The study was approved by the Regional Committees for Medical and Health Research Ethics Central Norway (REK 2012-1029). Furthermore, seven independent datasets of human GC from the TCGA database were used (Table 2).

**TABLE 1:** Demographic and clinical parameters of gastric cancer patients.

Number of patients		
Age group	49–53	1
	54–58	1
	59–63	2
	64–68	1
	69–73	2
	74–78	5
	79–83	3
	84+	1
Sex	Male	11
	Female	5
Pathologic characteristics		
Lauren classification	Intestinal	3
	Diffuse	4
	Mixed	2
	Not classified	7
Type of gastric resection	Total gastrectomy	7
	Subtotal gastrectomy	5
	Distal gastrectomy	4

**TABLE 2:** WNT/β-catenin signaling in human gastric cancer (one in the present study and 7 STAD datasets deposited in the TCGA database).

TCGA/Ingenuity Knowledge Base	WNT/β-catenin signaling		N (tumor samples)	N (control samples)
	*Z*-score	−log_10_(*p*)		
Human gastric cancer (the present study)	1.604	1.86E00	24	37
GSE48433; 354-stomach cancer [stomach] NA 3485 (PMID: 24885658) Hollingshead et al. (2014)	0.728	N/A	5	5
GSE48433; 171-stomach cancer [stomach] NA 3282 (PMID: 24885658) Hollingshead et al. (2014)	1.155	1.64E00	5	5
GSE118897; 1- stomach cancer [stomach] NA 628 (PMID: 30404039) Yang et al. (2019)	2.121	1.45E00	10	10
1-gastric adenocarcinoma (STAD) [stomach] NA 4052 Ingenuity Knowledge Base	2.138	2.29E00	70	36
10-gastric adenocarcinoma (STAD) [stomach] NA 4053 Ingenuity Knowledge Base	1.342	0	16	71
102-gastric adenocarcinoma (STAD) [stomach] NA 4056 Ingenuity Knowledge Base	1.134	0	20	71
111-gastric adenocarcinoma (STAD) [stomach] NA 4066 Ingenuity Knowledge Base	0.447	0	21	71

The mouse model of GC, i.e., the transgenic INS-GAS mice which spontaneously develop gastric cancer, was used (Zhao et al., 2014). Stomachs were collected from 26 mice, i.e., six females and four males INS-GAS mice at age of 15 months and eight females and eight males wild-type (WT) mice at age of 12 months, for gene expression profiling. In addition, 31 INS-GAS mice, i.e., 12 females and nine males at age of 10 months, and 10 WT mice, i.e., 6 females and four males at age of 10 months, were used for *in vivo* testing.

Animals were housed as four to five mice per cage on wood chip bedding with a 12 h light/dark cycle in a specific pathogen free environment with room temperature of 22°C and 40–60% relative humidity. Animals were inspected daily by investigators and authorized veterinarian using a scoring sheet. Animals should be euthanized at score of 10 if they are emaciated, underconditioned in five consecutive days, or show poor clinical signs (e.g., body weight, appearance, and behavior) before end of the study. This was done according to the Directive 2010/63/EU in which human primary endpoint are defined as “the earliest indicator in an animal experiment of (potential) pain and/or distress that, within the context of moral justification and scientific endpoints to be met, can be used to avoid or limit pain and/or distress by taking actions such as humane killing or terminating or alleviating the pain and distress (Hendriksen and Morton, 1999)” (https://www.humane-endpoints.info/en/council-directive-2010-63-eu). The study was approved by The Norwegian Food Safety Authority (Mattilsynet).

### Transcriptomics

Total RNA was extracted from the surgical biopsies of patients and harvested stomachs of mice. RNA quality and quantity were obtained using NanoDrop One (Thermo Scientific, Norway) and Agilent Bioanalyser. For human samples, RNA microarray of GC samples, including 24 tumors of intestinal, diffuse and mixed types from seven patients and 37 normal tissue from six patients, was performed using Illumina platform as described earlier (Zhao et al., 2014). Illumina microarray data was analyzed using Lumi on the log_2_ scale and analyzed using the empirical Bayesian method implemented in Limma. The data is accessible via Mendeley Data repository with DOI link at http://dx.doi.org/10.17632/hzmfshy7hp.1. Illumina identifiers (ILMN) were uploaded to Ingenuity Pathway Analysis (IPA, QIAGEN, Hilden, Germany) together with log_2_-fold change, *p*-values and *q*-values (false discovery rates). For mouse samples, RNA sequencing was performed using Illumina HiSeqNS500 instrument (NextSeq 500) at 75 bp with paired end (PE) reads using NS500H flow cells with 25M reads/sample. Paired end forward read length (R1): 81, reverse read length (R2): 81. Downstream processing and analysis of the data was performed in the Bioconductor environment in R. For humans, a total of 47,323 transcripts was assigned to analysis in which 37,489 transcripts were mapped and 9,834 transcripts unmapped by Ingenuity Pathway Analysis (IPA) (QIAGEN, Hilden, Germany). For mice, a total of 54,460 transcripts was loaded in which 54,162 were mapped/298 transcripts were unmapped in IPA. For mouse GC after ivermectin treatment, 54,416 transcripts were loaded in which all were mapped in IPA. Filtering of datasets included species (mouse or human) and *p*-value cut-off (*p* < 0.05). Gene expression was analyzed using a *t*-test between tumor and normal tissue in patients, between INS-GAS and WT mice and between INS-GAS mice with and without ivermectin. Genes with a *p*-value of less than 0.05 were considered to be differentially expressed. Transcriptomics datasets were analyzed using IPA. Molecular networks and canonical pathways were algorithmically constructed based on known connectivity and relationships among genes/proteins/metabolites using Ingenuity Knowledge Base. Local and regulatory *z*-scores for canonical pathways and diseases and biofunctions that overlapped with the experimental data of the present study were calculated using the formula described previously (Sitarz et al., 2018). IPA has sophisticated algorithms to calculate predicted functional activation/inhibition of canonical pathways, diseases and functions, transcription regulators and regulators based on their downstream molecule expressions (QIAGEN Inc., https://www.qiagenbioinformatics.com/products/ingenuitypathway-analysis). Fischer’s exact test was used to calculate a *p*-value determining the probability that the association between the genes in the datasets from human GC and mouse GC and the canonical pathway or disease/function by chance alone.

### Connectivity Map and Data/Pathway Mining

The concept of a Connectivity Map (cMap) was recently developed, whereby genes, drugs, and disease states are connected by virtue of common gene expression signatures (Qu and Rajpal, 2012; Subramanian et al., 2017; Musa et al., 2018). To identify candidate drugs, the gene expression signature of GC was generated based on the gene expression profile of human GC. A positive cMap score indicates there is a positive similarity between a given perturbagen’s signature, i.e., genes that are increased by treatment (in reference datasets) are also upregulated in the human GC dataset, while a negative score indicates that the two signatures are opposing. cMap was performed using the gene expression signature of human GC (*n* = 7 GC vs. *n* = 6 normal tissue).

Data mining was performed using the gene expression profile data of 61 samples from 16 patients, 26 samples from 26 mice, and 324 samples from seven independent datasets from the TCGA database. Furthermore, knowledge-based pathway mining was used based on previous studies that showed WNT/β-catenin signaling pathway as one of the important pathways in gastric tumorigenesis (Zhao et al., 2014; Rabben et al., 2021). Custom-made molecular networks were generated using the Ingenuity Knowledge Base. Networks were then algorithmically generated based on their interrelationships. WNT/β-catenin signaling pathway was constructed based on the transcriptomic data of INS-GAS mice and were then entered into the “Pathway” module of the IPA to obtain the nodes in every corresponding signaling pathway. Nodes from pathways were added as entries into the “My list”-function and all entries in the list were added to the “My pathway” in IPA. My pathway was used to produce a network of nodes/genes from the WNT/β-catenin signaling pathway that matched with our experimental data from INS-GAS vs. WT mice. The build-tool was used to connect nodes using edges, i.e., relationships including both direct and indirect interactions like chemical-protein interactions, ubiquitination, molecular cleavage, translocation, localization, phosphorylation, expression, protein-protein interactions, activation, regulation of binding, inhibition, membership, reaction, protein-DNA interactions, transcription and modification. The Canonical Pathway overlay-tool was used to arrange the entries into clusters based on pathway. Local *z*-scores were calculated in IPA based on the dataset’s correlation with the activated state. Negative *z*-scores indicate a decrease in activity, positive *z*-scores indicate an increase in activity. Canonical pathways were identified using statistical cut-offs at *p* < 0.05.

### *In Silico* Testing

The expression data from mouse GC was compared to all genes in the pathway. The molecule activity predictor (MAP)-function was used to predict activation/inhibition between the nodes in the network. The *in silico* tool integrated with the MAP-function was employed to predict effects on the network after gene inhibition and/or stimulation in the ivermectin cluster. Connections between genes were then algorithmically generated based on their interrelationships including both direct and indirect interactions like chemical-protein interactions, ubiquitination, molecular cleavage, translocation, localization, phosphorylation, expression, protein-protein interactions, activation, regulation of binding, inhibition, membership, reaction, protein-DNA interactions, transcription and modification. Network clusters of WNT/β-catenin pathway was constructed based on the transcriptomic data of INS-GAS mice (i.e., limited to and built on genes from the dataset). The build-tool was used to connect nodes using edges, i.e., relationships. Categorical values were set to each gene/node using a semi-quantitative method to quantify the color-change resulting from *in silico* inhibition. Local *z*-scores were calculated in IPA based on the dataset’s correlation with the activated state. Negative *z*-scores indicate a decrease in activity, positive *z*-scores indicate an increase in activity. Canonical pathways were identified using statistical cut-offs at *p* < 0.05.

### *In Vitro* Testing

GC cell lines included human gastric cancer cells MKN74 (intestinal type) and KATO-III (diffuse type) (for detailed information on molecular characteristic, see Yokozaki, 2000). It should be noticed that both cell lines overexpress β-catenin (Asciutti et al., 2011). Cells were maintained in RPMI-1640 medium (Sigma Aldrich, Oslo, Norway) supplemented with fetal bovine serum (10%, FBS), Sodium pyruvate and penicillin streptomycin solution (1%) (Sigma Aldrich, Oslo, Norway) in a humidified incubator holding 5% CO_2_ and 37°C. For proliferation assay, MKN74 and KATO-III were seeded in 96-well plates (2,500 cells/well and 3,000 cells/well, respectively) and incubated overnight. Ivermectin (MW: 875.09 g/mol) was dissolved in DMSO (100%) to 50 mM stock solution. Cells were treated with ivermectin (0–50 µM) or vehicle control (0.45% v/v DMSO) for 24, 48, and 72 h. Proliferation was measured using a commercial CCK-8 Kit (Sigma Aldrich, Oslo, Norway) with absorbance read at 450 nm. For cell cycle analysis, KATO-III cells were seeded as 3.0 × 10^5^ cells/well in 6-well plates and incubated for 72 h with medium change after 48 h. Ivermectin was added to the wells as final concentrations of 12, 15 or 18 µM for 24 h. Cells were harvested by trypsin, washed twice in room tempered PBS, resuspended in ice cold ethanol (70%) and kept at −20°C for minimum 15 min. Cells were washed twice in cold PBS and centrifuged (1,500 rpm, 5 min, 4°C), and resuspended in freshly prepared PI staining solution (0.25% Triton- X-100, 50 μg/ml propidium iodide (PI) and 200 μg/ml RNAase) for minimum 30 min. Cell cycle distribution was analyzed using FACS. Single cells were gated to exclude doublets and clustered cells. 2.0 × 10^4^ cells were counted per sample, and percentage cell distribution was derived from obtained histograms in the FACSDiva software. Results are presented as means of *n* = 3 replicates/treatments. Data was analyzed using Microsoft Excel 2010.

### *In Vivo* Testing

Thirty-one INS-GAS mice were randomly divided into two groups: ivermectin treatment (12 females and nine males at age of 10 months) and controls (no treatment, six females and four males at age of 10 months). Ivermectin was reconstituted from lyophilized powder in DMSO to 50 mM solution and then diluted in saline before use. The treatment regimen was designed to let the mice tolerate the procedure easily, i.e., intraperitoneal injection at a dose of 10 mg/kg in a volume of about 0.5 ml/mouse with 27G needle once per day for 5 days, followed by no treatment for 5 days and then injection once per day for 10 days. This regimen was repeated 10 days later. The total duration of treatment was 2 months (2 × 30 days). Vehicle treatment was not performed because neither vehicle *per se* nor procedure would lead to any significant stress response. The mice were euthanized under isoflurane inhalation anesthesia (2–3%), and stomachs were collected as described previously (Zhao et al., 2014). Tumor volume density (% of glandular area of the stomach occupied by tumor) was measured using a point count method (Zhao et al., 2014). The tissue samples were collected for transcriptomics as aforementioned.

### Statistical Analysis

Values are expressed as means ± SEM or SD (stated in individual figure legend). For comparison of two independent groups, student independent *t*-test was used. For comparison of multiple groups, one-way ANOVA with Tukey’s or Dunnett’s post-hoc tests were used (stated in individual figure legend). SPSS version 26.0 for Windows (SPSS Inc., Chicago, IL, United States) was used and a *p*-value < 0.05 was considered to be statistically significant. Other methods are stated in corresponding figure legends.

## Results

### Computational Drug Repositioning Suggests the WNT/β-Catenin Signaling as Potential Target of Ivermectin

**FIGURE 2:**
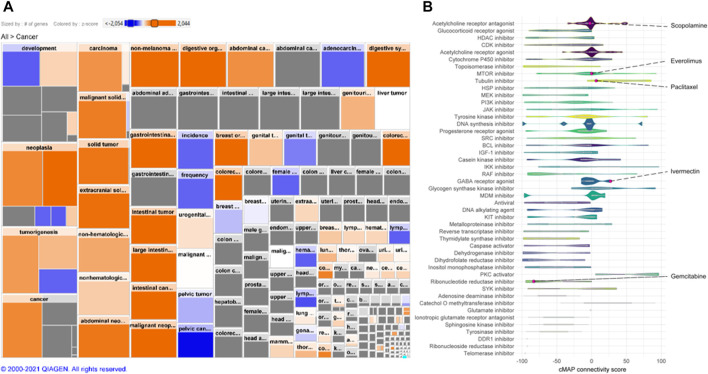
Gene expression signature and connectivity map (cMAP). **(A)** Heatmap of human GC gene expression signature that constitutes an activation of cancer disease based on differential expression of >22,000 genes. Size of square is proportional to the number of genes contained in the specific function and color represent activity state (*z*-score; orange: activated, blue: decreased). **(B)** Connectivity map (cMap) showing associations between a large-scale compendium of functional perturbations in cancer cell lines coupled to the human GC gene expression signature based on the L1000 assay (Subramanian et al., 2017). Note: Ivermectin and other known drugs are visualized.

A cMap was created according to the gene expression signature (Figures 2A,B). A total of 2,428 drugs were categorized into 47 groups of inhibitors for, e.g., DNA synthesis, murine double minute (MDM) and lactate dehydrogenase, including ivermectin. Additionally, drugs we have demonstrated previously, including gemcitabine, paclitaxel, everolimus, and scopolamine, were also found (Zhao et al., 2014; Rabben et al., 2021).

**FIGURE 3:**
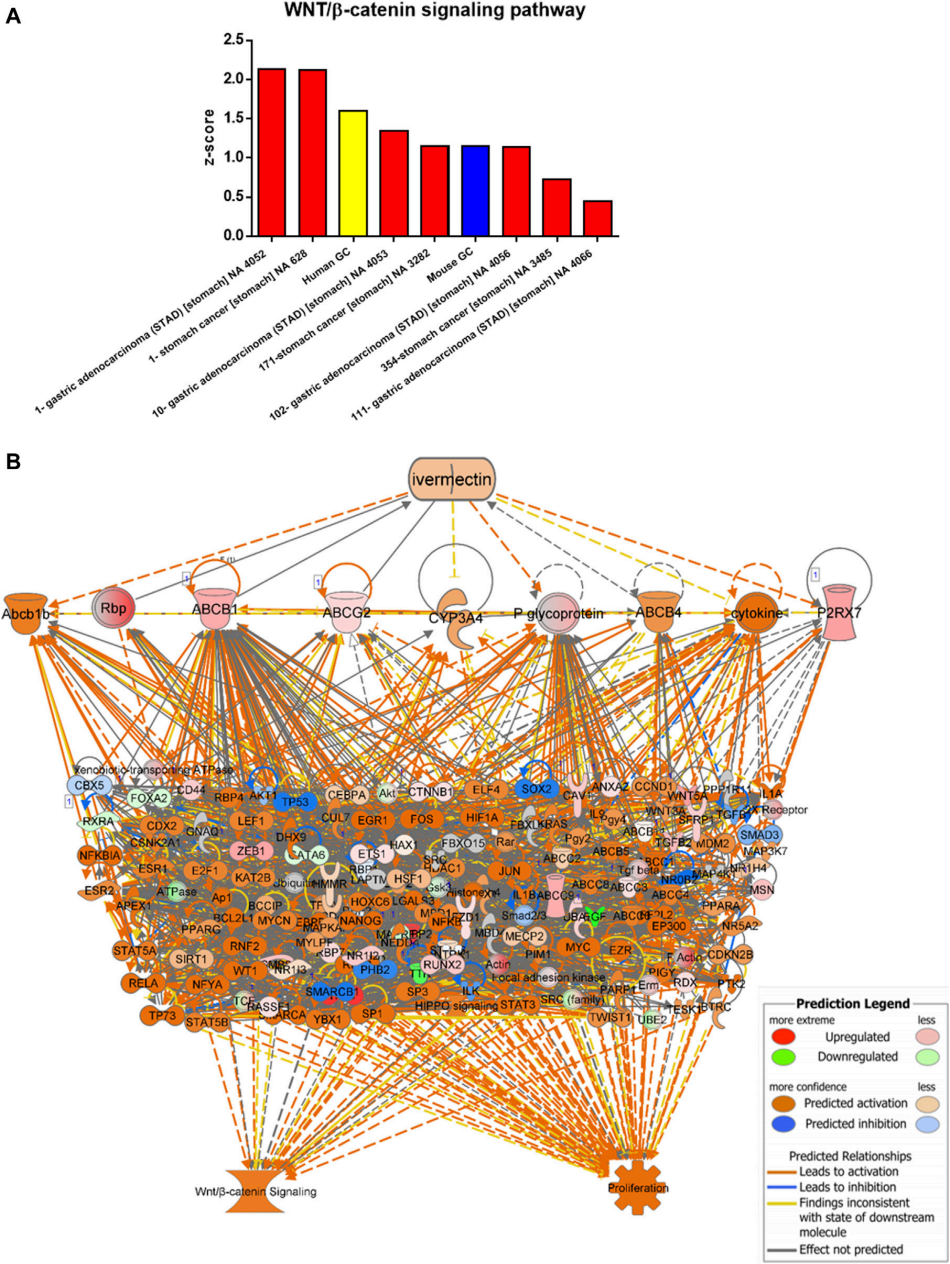
Data/pathway mining of WNT/β-catenin signaling pathway. **(A)** Activation of WNT/β-catenin signaling pathway in eight datasets of human GC (including one used in the present study as indicated in yellow) and one dataset of mouse GC (in blue). **(B)** Hierarchical network representation showing ivermectin and drug targets with downstream signaling pathways of WNT/β-catenin and proliferation. The schema was created in IPA using the grow-tool and the Ingenuity Knowledge Base. The molecular entities (genes and proteins) as well as molecular functions and interactive networks were connected based on interrelationships identified by the Ingenuity Knowledge Base. Expression levels from mouse GC vs. WT. *p* < 0.05. See also Table 3 *In silico* testing shows ivermectin inhibits WNT/β-catenin signaling.

Data/pathway mining revealed activation of the WNT/β-catenin signaling in human as well as mouse GC (Table 2). In addition to our own human GC and mouse GC data, seven independent datasets of human stomach adenocarcinoma (STAD) were found to have activation of the WNT/β-catenin (Figure 3A and Table 2). Using the knowledge-based repositioning strategy, nine targets were identified in connection with WNT/β-catenin signaling pathway and proliferation in both human and mouse GC (Figure 3B), i.e., ATP-dependent translocase (Abcb1b), retinol-binding proteins (Rbp), ATP binding cassette subfamily B member 1 (ABCB1), ATP Binding cassette subfamily G member 2 (ABCG2), cytochrome P450 family 3 subfamily A member 4 (CYP3A4), P-glycoprotein (also known as multi-drug resistant protein, MDRP), ATP binding cassette subfamily B member 4 (ABCB4), cytokine, and P2X purinoceptor 7 (P2RX7). Each gene/protein connected to a subset of algorithmically chosen genes based on the Ingenuity Knowledge Base. These genes were collectively activating both WNT/β-catenin signaling and proliferation, resulting in locally activated *z*-scores (shown in orange) (Table 3).

**TABLE 3:** Gene expression in networks comprised of ivermectin interactions and WNT/β-catenin signaling pathways in mouse gastric cancer (as presented in Figure 4A).

Gene	Ensembl ID	Log_2_ FC	*p*-value	Entrez gene ID for patients	Entrez gene ID for mice
ABCB1	ENSMUSG00000040584	1.877	2.04E-04	5243	18671
ABCC3	ENSMUSG00000020865	0.525	1.16E-02	8714	76408
ABCC9	ENSMUSG00000030249	1.835	2.74E-11	10060	20928
ABCG2	ENSMUSG00000029802	0.921	3.97E-03	9429	26357
ANXA2	ENSMUSG00000032231	0.679	3.38E-04	302	12306
CAV1	ENSMUSG00000007655	1.181	8.61E-04	857	12389
CD44	ENSMUSG00000005087	1.156	1.42E-05	960	12505
CRABP2	ENSMUSG00000004885	4.298	2.97E-02	1382	12904
CTNNB1	ENSMUSG00000006932	0.208	4.75E-02	1499	12387
EGF	ENSMUSG00000028017	−1.946	3.86E-03	1950	13645
ETS1	ENSMUSG00000032035	0.621	2.69E-02	2113	23871
FBXL13	ENSMUSG00000048520	3.359	3.15E-02	222235	320118
FOXA2	ENSMUSG00000037025	−0.351	1.45E-02	3170	15376
FZD1	ENSMUSG00000044674	0.670	1.76E-03	8321	14362
GATA6	ENSMUSG00000005836	−0.331	1.95E-02	2627	14465
IL1A	ENSMUSG00000027399	3.560	1.60E-02	3552	16175
MAPRE1	ENSMUSG00000027479	−0.456	5.95E-03	22919	13589
MECP2	ENSMUSG00000031393	−0.374	5.89E-02	4204	17257
MSN	ENSMUSG00000031207	1.383	1.79E-17	4478	17698
NR1I2	ENSMUSG00000022809	1.174	2.29E-08	8856	18171
P2RX7	ENSMUSG00000029468	2.097	3.93E-07	5027	18439
PSMB8	ENSMUSG00000024338	1.376	8.55E-03	5696	16913
RASSF1	ENSMUSG00000010067	0.291	4.88E-02	11186	56289
RBP7	ENSMUSG00000028996	0.851	4.24E-02	116362	63954
RDX	ENSMUSG00000032050	0.749	1.53E-03	5962	19684
RUNX2	ENSMUSG00000039153	1.241	2.32E-04	860	12393
RXRA	ENSMUSG00000015846	−0.404	2.74E-03	6256	20181
SFRP1	ENSMUSG00000031548	1.093	2.91E-03	6422	20377
TGFB2	ENSMUSG00000039239	1.573	3.53E-05	7042	21808
TP63	ENSMUSG00000022510	5.030	2.28E-02	8626	22061
TTR	ENSMUSG00000061808	−1.516	1.78E-04	7276	22139
UBA7	ENSMUSG00000032596	1.008	2.12E-03	7318	74153
WNT5A	ENSMUSG00000021994	2.170	1.90E-14	7474	22418
ZEB1	ENSMUSG00000024238	2.031	8.00E-12	6935	21417

**FIGURE 4:**
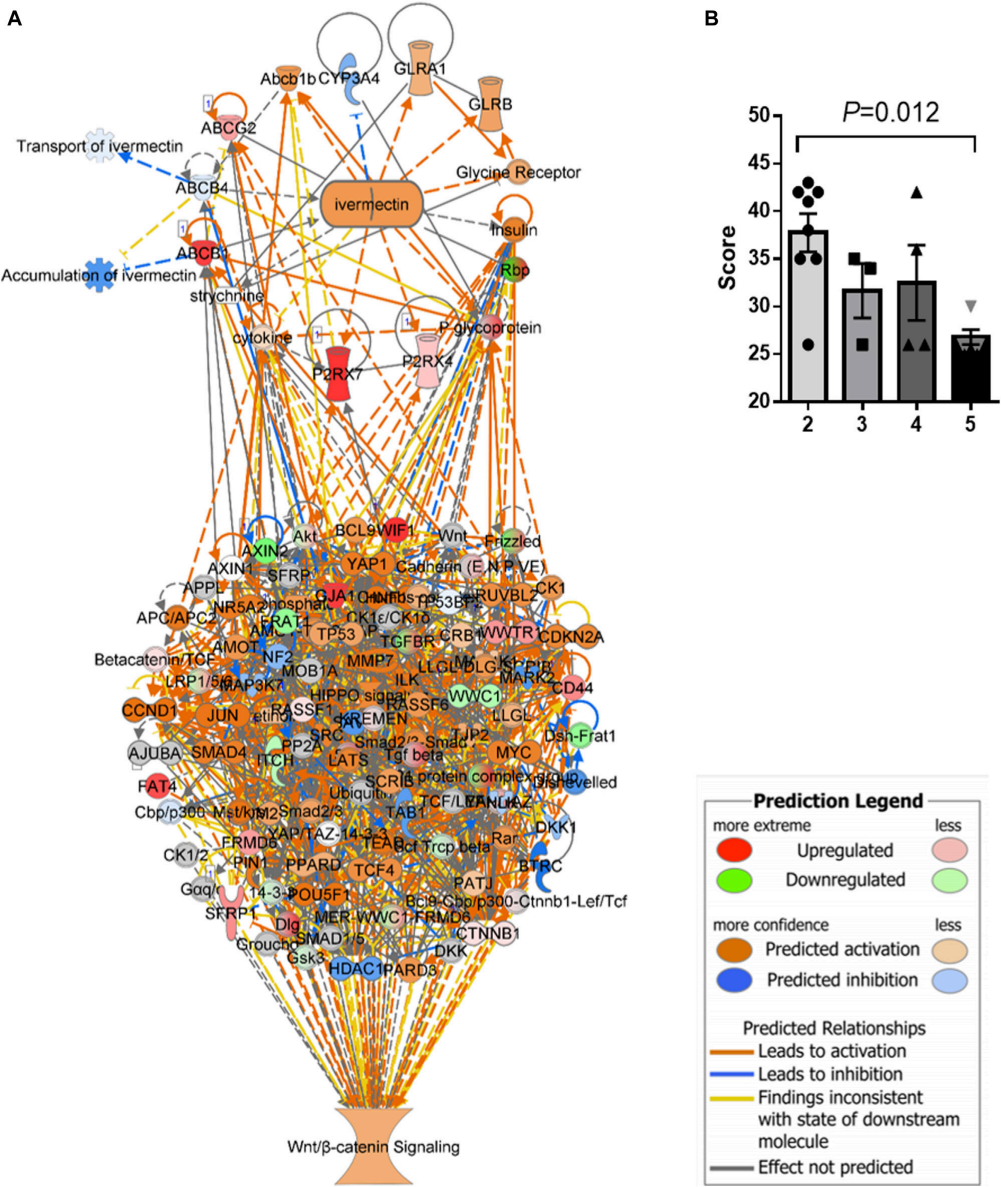
*In silico* interaction network of ivermectin with WNT/β-catenin signaling pathway. **(A)** An interaction network of ivermectin with WNT/β-catenin signaling pathway was made using the Ingenuity Knowledge Base. **(B)** Two-/three-/four-/five combinations of inhibition/stimulation of nodes in the ivermectin cluster (**A**, upper cluster) had “dose-dependent” effect of the sum network scores of WNT/β-catenin (including HIPPO) pathway (**A**, lower cluster). Categorical values were set to each gene/node using a semi-quantitative method to quantify the color-change resulting from *in silico* inhibition. Dark blue colored nodes were represented by − 2, light blue as − 1, white as 0, light orange as +1 and dark orange as +2. Values are represented of means of *n* = 3–8 experiments per node/gene. Multiple comparison using one-way ANOVA with Tukey post hoc test was used. Created in IPA (QIAGEN).

An *in silico* interaction network of ivermectin with WNT/β-catenin signaling pathway was constructed (Figure 4A). Inhibition of the targets of ivermectin led to the inhibition of downstream nodes in a “dose-dependent manner” (Figure 4B). It should be noticed that the inhibition of single molecules was not enough to have inhibitory or stimulatory effects on the signaling pathway.

### Ivermectin Inhibits Cell Proliferation and Induces Cell Cycle Arrest

**FIGURE 5:**
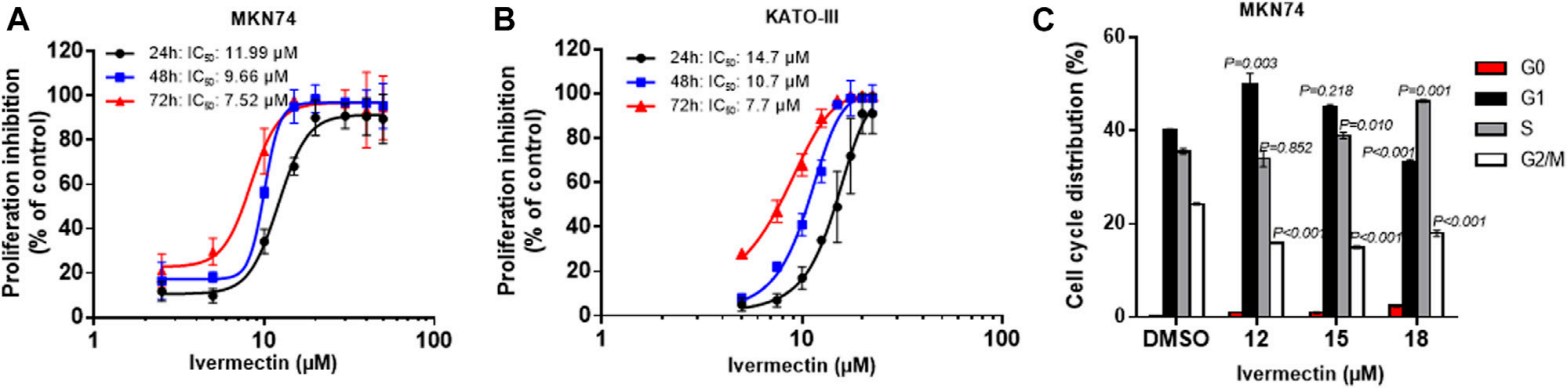
Time- and concentration-dependent effects of ivermectin on proliferation and cell cycle. **(A,B)** Concentration-response curves upon 24–72 h ivermectin-treatment in MKN74 and KATO-III cells. Proliferation was assessed using CCK-8 Kit at 450 nm. IC_50_ values were calculated from sigmoidal regression curve fitting using variable slope on normalized response from log_10_-transformed x-values (GraphPad Prism v.6). **(C)** Cell cycle analysis of KATO-III cells. Means of *n* = 3 replicates/treatment with SD. One-way ANOVA with Dunnett’s post hoc test (2-sided) compared to respective control groups.

Testing of ivermectin in MKN74 and KATO-III cells showed that there were time- and concentration-depended inhibitions of proliferation by the drug with similar IC_50_ values for the periods of 24, 48 and 72 h (Figures 5A,B). Furthermore, ivermectin induced cell cycle arrest in a concentration-depended manner (Figure 5C). It should be noticed that ivermectin at IC_50_ did not affect the cells in S-phase but increased percentage of cells in G_1_ while reducing percentage of cells in G_2_/M phases. By contrast, higher concentration of ivermectin increased percentage of cells in S phase while reducing the percentage of cells in G_1_ and G_2_/M phases, suggesting that ivermectin arrested cells at the G_1_ phase at IC_50_ and higher dose of the drug shifted cells to S phase.

### Ivermectin Reduces Tumor Size Which Was Associated With Inactivation of WNT/β-Catenin Signaling, Down Regulation of Cell Proliferation and Upregulation of Cell Death Signaling Networks

**FIGURE 6:**
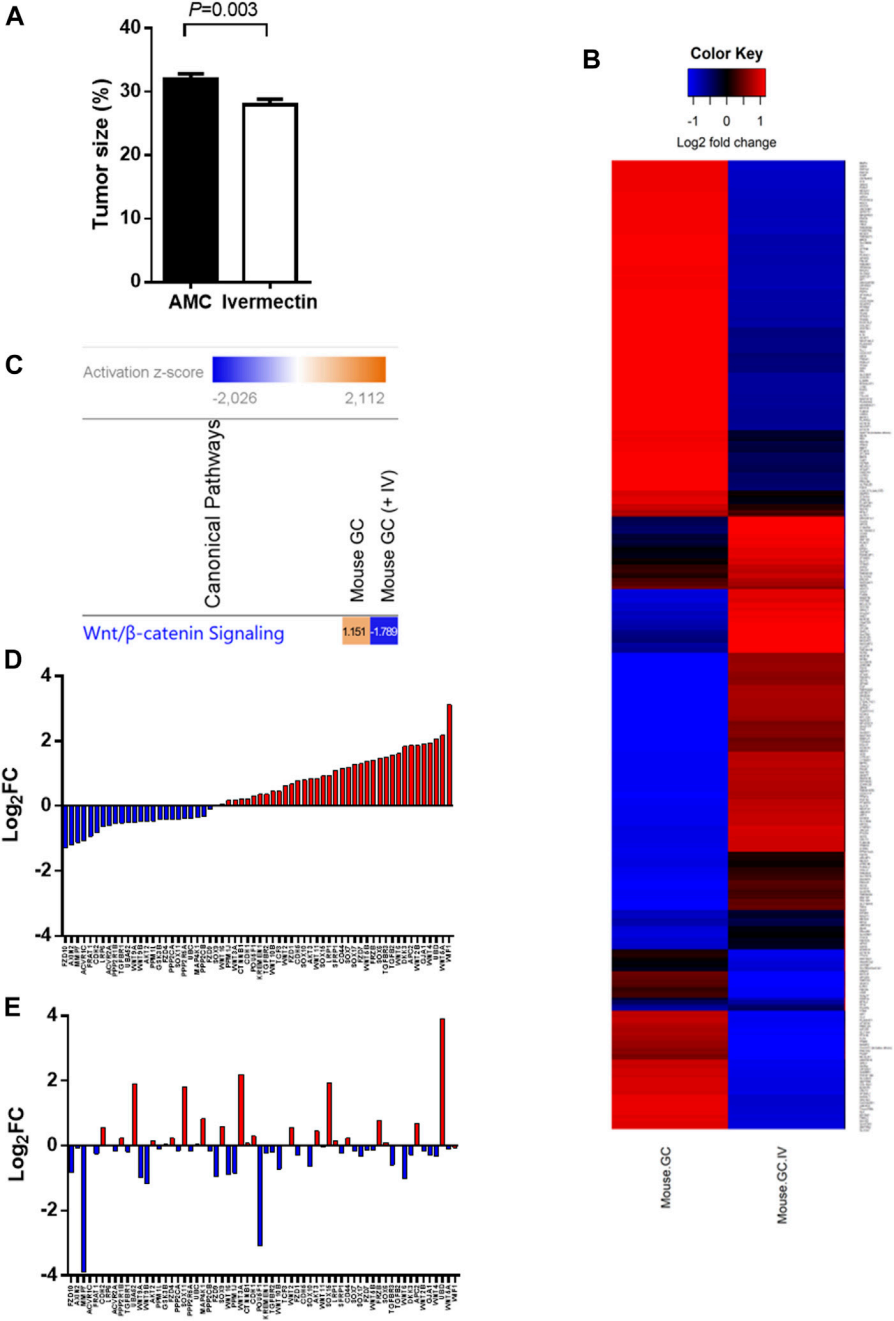
Tumor size and gene expression profiles in response to ivermectin. **(A)** Tumor size (% of glandular area of stomach occupied by tumor) in age-matched controls (AMC, *n* = 14) and ivermectin-treated mice (*n* = 17) (Ivermectin). Independent t-test (2-sided) between group means (normality assumption met). Error bars represent SEM. **(B)** Global gene expression profile of mouse GC with and without ivermectin treatment (created in RStudio using heatmap.2 function). Only differentially expressed genes with *p* < 0.05 are included (4,112 genes). **(C)** WNT/β-catenin pathway was activated (*z*-score = 1.151) in mouse GC without treatment but inhibited in mouse GC with ivermectin treatment (*z*-score = −1.789). **(D,E)** WNT/β-catenin gene expressions in mouse GC mice without treatment **(D)** and with ivermectin treatment **(E)**. Note: same orders of individual genes in **(D)** and **(E)**.

A treatment regimen using ivermectin at 10 mg/kg for 2 months was established based on the *in silico* and pilot experiments. Mice tolerated the treatment well, although some mice had weight loss during treatment (<15%, *p* > 0.05, two-tailed). The mice had no serious side effects of ivermectin and no mice that were treated with ivermectin were killed according to the human primary endpoints which include stressful behavior, abdominal pain and impaired physical activity. The tumor size was reduced by ivermectin treatment (Figure 6A). Comparison analysis between mouse GC with and without ivermectin treatment revealed 4,112 differentially expressed genes (Figure 6B). The genes involved in WNT/β-catenin signaling pathway were particularly inhibited by ivermectin treatment, as shown by a change in *z*-scores from 1.151 (mouse GC without treatment) down to −1.789 (mouse GC after ivermectin treatment) (Figure 6C and Table 2) and log_2_ fold-changes (Figure 6D vs. 6E).

**FIGURE 7:**
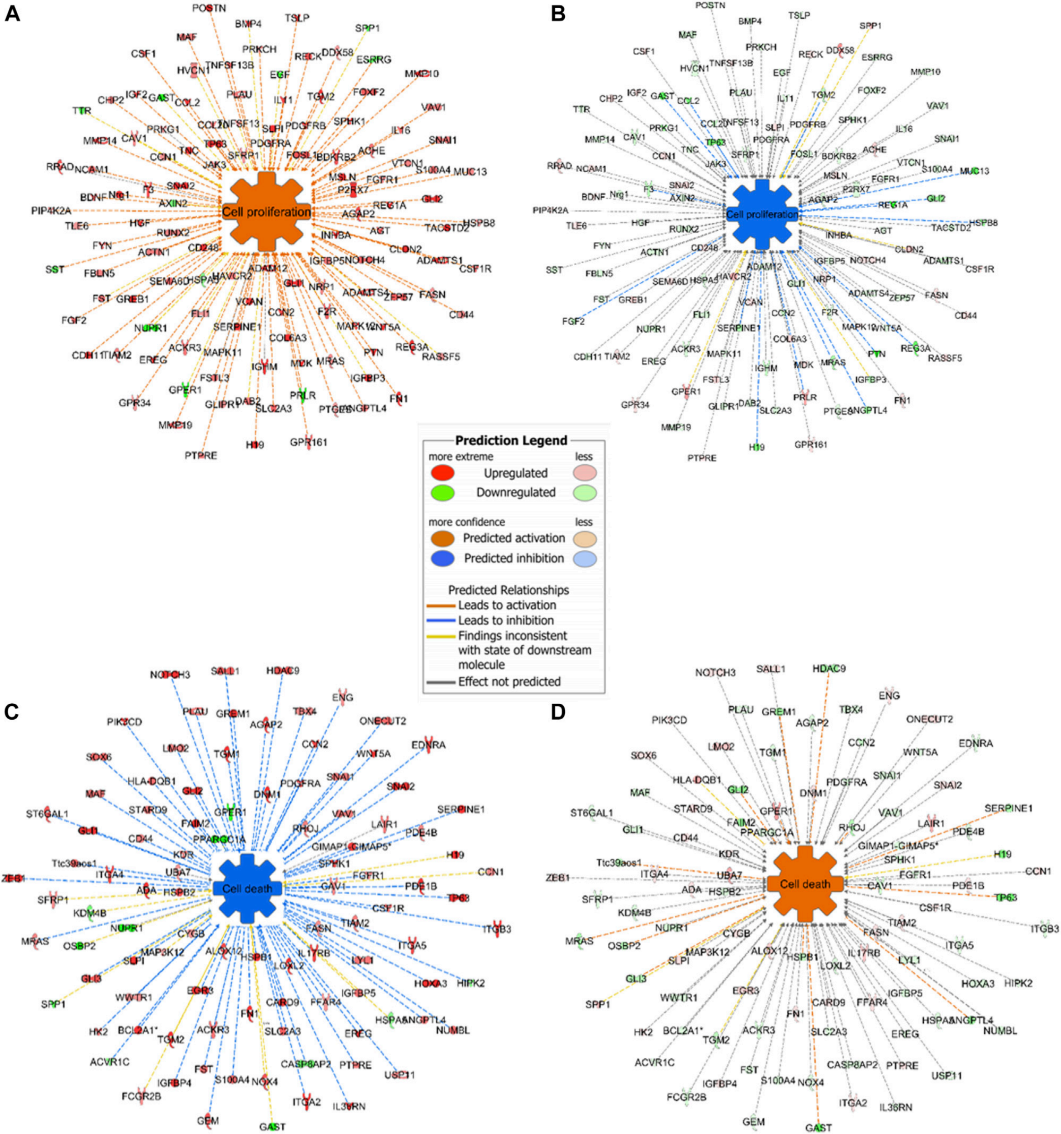
Representative networks of cell proliferation and cell death in mice with or without ivermectin treatment. **(A)** Mouse GC cell proliferation network was created using BioProfiler in IPA. Only genes differentially expressed (*p* < 0.05, Log_2_FC ± 1.0) between mouse GC vs. WT were included. Overexpression of genes in the network results in activation of cell proliferation (orange; *z*-score > 0). **(B)** Genes in the cell proliferation network was downregulated in GC mice with ivermectin treatment (blue; *z*-score < 0). **(C)** Mouse GC cell death network including apoptotic markers was created using BioProfiler in IPA. Only genes differentially expressed (*p* < 0.05, Log_2_FC ± 1.0) between mouse GC vs. WT were included. Overexpression of genes in the network results in inhibition of cell death (blue; *z*-score < 0). **(D)** Cell death network was aberrantly expressed in GC mice with ivermectin treatment (orange; *z*-score > 0).

Expression analysis in IPA revealed that cell proliferation was activated in mouse GC without treatment and inactivated in mouse GC with treatment. On the other hand, cell death including apoptosis was inactivated in mouse GC without treatment but activated in mouse GC with treatment (Figures 7A–D).

## Discussion

The next generation connectivity map (cMap) has been recently developed and should be acknowledged that the cMap methods and data are available without restriction to the research community (Subramanian et al., 2017). As pointed out in the original paper, a future comprehensive cMap might expand in multiple dimensions, e.g., new cell types, patient-derived induced pluripotent stem cells and genome-edited isogenic cell lines (Subramanian et al., 2017). Using this method, we found that the scores of the known drugs in treatment of GC (including ivermectin) were too low to indicate strong associations between these drugs and human GC gene expression signature, which was most likely due to the fact that the reference profile catalogue of cMap has been built to date on 12,328 genes of various cancer cell lines (including AGS which is a moderately differentiated human gastric adenocarcinoma hyperdiploid cell line) but not tumor tissues (https://clue.io/connectopedia/l1000_gene_space and https://clue.io/connectopedia/core_cmap_cell_panel).

In addition to the hypothesis generation approach by cMap, we further utilized data mining and pathway mining of knowledge-based datasets to identify the potential drugs in connection with a broad concept ranging from molecular entities (such as genes and proteins) to biological phenomena (such as molecular functions, pathways and phenotypes). Based on a better understanding of GC biology and signaling pathways, in the present study we focused on the WNT/β-catenin pathway by utilizing the algorithms of IPA which is built on a comprehensive, manually curated content of the QIAGEN Knowledge Base (over 57,000 publicly available datasets and continuously updated).

The results of the present study showed that the potential molecular targets and the mechanisms of action of ivermectin in GC differed from those in parasites in which ivermectin causes an influx of Cl-ions through the cell membrane of invertebrates by activation of specific ivermectin-sensitive ion channels (Laing et al., 2017; Chen and Kubo, 2018). In the present study, we identified ivermectin in connection with cell proliferation, particularly towards the genes (e.g., members of the adenosine triphosphate (ATP)-binding cassette (ABC) transporters). ABC are a superfamily of membrane proteins which play significant roles in transporting various exogenous and endogenous substances across membranes against concentration gradients through ATP hydrolysis, and many of these transporters are known as multidrug resistance proteins (MRPs) (Mao et al., 2019). As showed in the present study, ivermectin also acted on the ABC and the signaling pathways, leading to inhibition of cell proliferation by deactivating LXR/RXR signaling (Beltowski, 2011; Saito-Hakoda et al., 2015).

It has been shown that activation of the WNT/β-catenin signaling pathway plays a pivotal role in many types of cancer (Clevers, 2006; Zhan et al., 2017). Previously, we and other research groups have demonstrated that the tumorigenesis of gastric cancer involves the WNT/β-catenin signaling pathway and the inhibition of the signaling pathways by means of denervation can suppress the tumorigenesis (Zhao et al., 2014; Chiurillo, 2015; Koushyar et al., 2020; Rabben et al., 2021). In the present study, we applied *in silico* modelling to show that ivermectin could inhibit the WNT/β-catenin signaling pathway including HIPPO signaling pathway, which is known to interact each other (Hayakawa et al., 2017; Li et al., 2019). We then employed *in vitro* and *in vivo* approaches to show that ivermectin could inhibit cell proliferation and reduce tumor size, which was associated with the inhibition of the WNT/β-catenin signaling pathway. Thus, we may suggest that ivermectin could target the WNT/β-catenin singling pathway, leading to a reduced tumorigenesis. This was also in line with possible antitumor actions of ivermectin in other types of cancer cells, such as breast, colon, lung, prostate and bladder (Melotti et al., 2014; Diao et al., 2019; Nappi et al., 2020).

Control of cell proliferation generally occurs during the G_1_ phase and multiple signals, ranging from growth factors to DNA damage to developmental cues, influence the decision to enter S phase, when DNA is replicated (Duronio and Xiong, 2013). The results of the present study showed that ivermectin altered cell cycle in a concentration-dependent manner, which is consistent with a previous report showing accumulation of cells in the G_1_/S phases (Zhang et al., 2019). In the present study, IC_50_-dose of ivermectin caused cell cycle arrest at G_1_ phase, whereas at higher doses, it caused S phase arrest. It has been suggested that WNT/β-catenin activation triggered cells in S phase, and HIPPO signaling might involve in G_1_ phase (Benham-Pyle et al., 2016; Kim et al., 2019). The evidence of possible link between the cell cycle arrest and inhibition of WNT/β-catenin and/or HIPPO singling pathways is needed to be further investigated, particularly in the context of ivermectin for GC.

There were several limitations of the present study. The cell proliferation and apoptosis in the *in vitro* experiment were not evaluated further by flow cytometry nor specific assays, e.g., annexin V staining or caspase activity. However, the gene expression profiling confirmed the association between the activities of networks of cell proliferation and cell death in mice, namely increased in cell proliferation and decrease in cell death in GC mice without treatment, and reversed activities in GC mice treated with ivermectin. It should be noticed that the decrease in tumor size 2 months after ivermectin treatment was modest. As a matter of fact, in a separate experiment, we found that chemotherapy with 5-FU and oxaliplatin at the maximal dosage given to GC mice at the same age as ones in this study was without inhibition on the tumor size during 2 months of treatment (as same as in this study) (data not shown). However, the impacts of ivermectin treatment after a longer period of treatment alone and/or in combination with chemotherapy on resistance, migration and invasion could be worthwhile for future investigation. The results of the present study showed evidence of possible involvement of WNT/β-catenin signaling pathway in connection with the anti-cancer effect of ivermectin. For instance, prediction of ivermectin was successfully made by the WNT/β-catenin signaling pathway mining but not cMap. Validation of ivermectin was significant *in silico* model of the WNT/β-catenin signaling pathway. Up-regulation of WNT/β-catenin signaling pathway took place in patients, human cell lines and mouse model of GC. Ivermectin treatment induced downregulation of the WNT/β-catenin signaling pathway in the mouse GC. However, additional evidence is needed to demonstrate that the effect of ivermectin is dependent on WNT/β-catenin signaling pathway. For instance, it would be worthwhile to further investigate how modulation of the WNT/β‐ catenin signaling pathway with specific inhibitors and activators will affect the response to ivermectin treatment *in vitro* and *in vivo*.

## Conclusions

The results of the present study show that ivermectin is a promising drug candidate for treatment of GC. The results may indicate an alternative mechanism of action of ivermectin, i.e., inhibition of the WNT/β-catenin signaling pathway in mammals rather than it acts on glutamate-gated chloride channels, which are common in nematodes, insects and ticks, thereby paralysing pharyngeal and somatic muscles. As ivermectin is exceptionally safe for mammals because of the blood/brain barrier, further pre-clinical and clinical studies of repositioning ivermectin for GC are warranted.

## Data Availability

The original contributions presented in the study are publicly available. This data can be found here: The mouse RNA seq/microarray data have been deposited in the NCBI Bioproject database under the accession number PRJNA690520 which can be accessed using the following link: http://www.ncbi.nlm.nih.gov/bioproject/690520. The human microarray data is available online via Mendeley Data repository with DOI link at http://dx.doi.org/10.17632/hzmfshy7hp.1. The RNAseq data in mouse GC after ivermectin treatment is available from the authors upon request.
